# Understanding cyber grooming in children and adolescents: a scoping review of risk factors and prevention strategies

**DOI:** 10.3389/fpsyt.2026.1896109

**Published:** 2026-07-23

**Authors:** Kenan Kaya, Ziyaettin Erdem

**Affiliations:** 1 Department of Forensic Medicine, Çukurova University, Adana, Türkiye; 2 Council of Forensic Medicine, Eskişehir Branch Directorate, Eskişehir, Türkiye

**Keywords:** adolescents, child grooming, child protection, cyber grooming, digital safety, online exploitation, online victimization, prevention strategies

## Abstract

The increasing digitalization of children’s daily lives has created new opportunities for communication, education, and social interaction while simultaneously increasing exposure to online exploitation risks. Among these risks, child grooming and cyber grooming have emerged as significant psychological, social, and public health concerns. Cyber grooming extends traditional grooming processes into digital environments through the use of anonymity, persistent communication, and technological accessibility, enabling offenders to manipulate and exploit children and adolescents in online settings. This scoping review aimed to examine the existing literature on grooming and cyber grooming, focusing on psychological, social, technological, and preventive dimensions. A literature search was conducted using PubMed, Scopus, and Web of Science databases for studies published between 2000 and 2025. Relevant findings were synthesized thematically to evaluate victim and perpetrator characteristics, developmental and environmental risk factors, prevention strategies, and legal approaches. The reviewed literature indicates that adolescents with high online engagement, limited digital literacy, emotional vulnerability, and inadequate social support may be at increased risk of grooming victimization. Offenders frequently utilize emotional manipulation, secrecy, and digital communication tools to establish trust and maintain control. Current evidence further suggests that effective prevention requires multidimensional approaches integrating digital literacy education, family involvement, psychological support, platform safety measures, and legal protections. Despite growing awareness of cyber grooming, important gaps remain regarding long-term psychological outcomes, prevalence variability, and the effectiveness of intervention programs. This review highlights cyber grooming as a multifaceted developmental and psychological phenomenon shaped by the interaction of individual vulnerabilities, online environments, and offender manipulation strategies. Effective prevention therefore requires coordinated, evidence-based, and multidisciplinary child protection efforts integrating psychological, technological, educational, and legal perspectives to improve children’s safety and well-being in digital environments.

## 1 Introduction

The proliferation of digital technologies has transformed childhood experiences, creating both opportunities and vulnerabilities. Children today engage with peers, educators, and broader communities through social media, online games, messaging applications, and content-sharing platforms. While these interactions foster learning, creativity, and social bonding, they also create avenues for exploitation, particularly by individuals who seek to manipulate children for sexual purposes. Grooming—long studied in psychology and criminology—has evolved alongside digital platforms, giving rise to cyber grooming, which is characterized by its reliance on online communication, anonymity, and technological reach (1).

Traditional grooming involves manipulative strategies designed to establish trust and emotional rapport with a child in order to facilitate sexual abuse. Perpetrators often exploit developmental vulnerabilities, including children’s emotional dependence, curiosity, and desire for social acceptance. Cyber grooming extends these dynamics into digital spaces, enabling offenders to interact with multiple victims simultaneously, maintain persistent contact, and employ multimedia content to deepen emotional attachment (2).

The consequences of grooming—whether offline or online—are profound. Victims may experience long-term psychological effects such as depression, anxiety, post-traumatic stress disorder, and social withdrawal. Academic performance and peer relationships are often negatively impacted, and the secrecy associated with grooming can delay disclosure and intervention (3). These findings highlight the importance of understanding both the psychological mechanisms underlying grooming behaviors and the technological affordances that facilitate cyber grooming.

Cyber grooming represents not only a behavioral phenomenon but also a public health and societal concern. It intersects with issues of child development, digital literacy, and platform design, emphasizing the need for multidisciplinary research and intervention strategies. Furthermore, the rapidly evolving landscape of social media, online gaming, and digital communication continues to create new challenges for parents, educators, policymakers, and law enforcement agencies.

Recent global evidence further highlights the magnitude of online child sexual exploitation and abuse (OCSEA). In a large-scale systematic review and meta-analysis including 123 studies, the pooled prevalence of overall OCSEA during the previous year was estimated at 8.1%, while online solicitation and non-consensual sexual image-related experiences affected approximately 12.5% and 12.6% of children, respectively (4). These findings underscore the widespread nature of online sexual victimization and the urgent need for effective prevention and intervention strategies.

Despite the growing body of literature on grooming and cyber grooming, there remains a need for integrative reviews that examine psychological, social, and technological dimensions within a unified framework. Therefore, this scoping review aims to map and synthesize the existing literature on grooming and cyber grooming in children and adolescents. Specifically, the review seeks to: (1) define and contextualize grooming and cyber grooming; (2) examine victim and perpetrator characteristics within psychological, social, and technological frameworks; and (3) evaluate prevention strategies, legal approaches, and current gaps in the literature. A comprehensive understanding of these dynamics is essential for developing effective interventions, improving digital safety awareness, and strengthening child protection policies in both offline and online environments.

## 2 Methods

This study was designed as a scoping review to examine the existing literature on child grooming and cyber grooming, with a particular focus on psychological, social, technological, and preventive dimensions. The review aimed to provide a broad overview of current knowledge, identify key themes, and explore existing gaps in the literature regarding victim and perpetrator characteristics, risk factors, prevention strategies, and emerging challenges associated with online grooming behaviors. The review was conducted in accordance with the Preferred Reporting Items for Systematic Reviews and Meta-Analyses Extension for Scoping Reviews (PRISMA-ScR) guidelines.

A comprehensive literature search was conducted using the PubMed, Scopus, and Web of Science databases. Relevant studies published between 2000 and 2025 were identified using combinations of the following keywords: “child grooming”, “cyber grooming”, “online grooming”, “online child sexual exploitation”, “child sexual abuse”, “online safety”, “digital literacy”, and “prevention strategies”. Additional relevant publications were identified through manual screening of reference lists from eligible articles.

Studies published in English and focusing on grooming or cyber grooming involving children and adolescents were considered eligible for inclusion. Original research articles, review articles, theoretical papers, and reports addressing psychological, social, technological, legal, or preventive aspects of grooming behaviors were included. Studies unrelated to child or adolescent populations, non-English publications, duplicate records, and publications lacking sufficient relevance to grooming-related behaviors were excluded.

**Figure 1:**
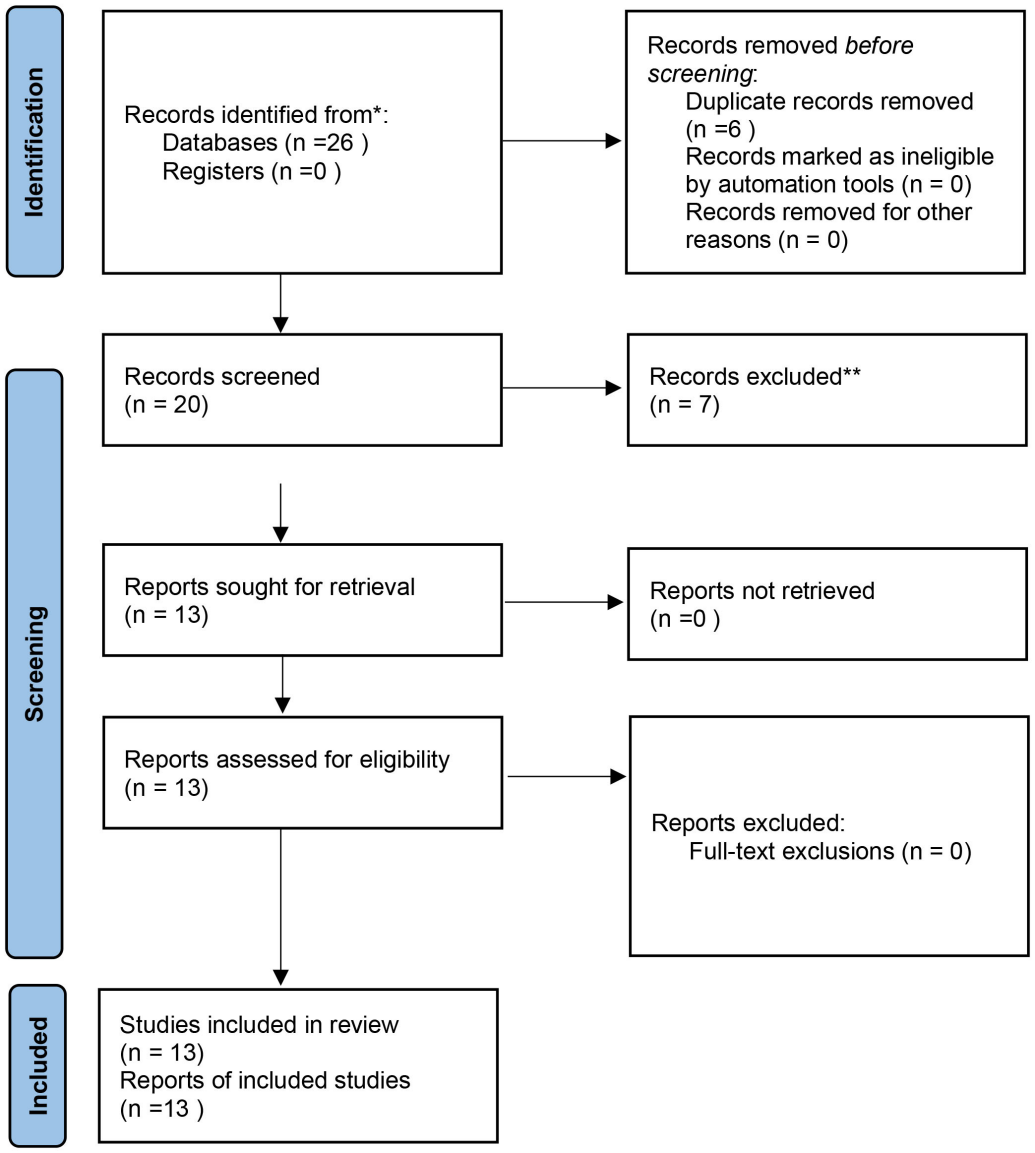
PRISMA-ScR flow diagram of study selection. Studies were synthesized thematically according to psychological, social, technological, legal, and preventive dimensions.

The database search yielded a total of 26 records (PubMed = 9, Scopus = 8, and Web of Science = 9). After the removal of six duplicate records, 20 records remained for title and abstract screening. Following the screening process, seven records were excluded due to insufficient relevance to the study objectives. The remaining 13 records were assessed through full-text review and formed the basis of the thematic synthesis. The study selection process is presented in the PRISMA-ScR flow diagram (**Figure 1**).

Data extraction focused on key study characteristics, including publication year, study design, sample characteristics, and principal findings related to grooming processes, victim vulnerability, perpetrator characteristics, technological factors, prevention strategies, and legal considerations. The included literature was examined descriptively, and findings were synthesized thematically according to these major domains.

Given the exploratory and integrative nature of the review, a qualitative narrative synthesis approach was adopted to summarize, compare, and interpret findings across the included studies. This approach facilitated the identification of recurring themes, patterns, and areas requiring further research within the field of child grooming and cyber grooming.

## 3 Grooming concept: historical and conceptual framework

Grooming has historically been conceptualized as a deliberate and strategic process through which an adult manipulates a child to gain sexual access. Craven et al. (5) describe grooming as a sequence of stages involving the identification of a vulnerable child, the establishment of trust and emotional rapport, the gradual introduction of sexual content, and eventual sexual exploitation. These stages exploit the inherent power imbalance between adults and minors and frequently rely on psychological manipulation strategies such as flattery, attention, gifts, and emotional reinforcement.

From a psychological perspective, grooming primarily targets developmental vulnerabilities. Children may perceive attention and emotional engagement as genuine affection, thereby reducing their ability to recognize manipulative intent. This vulnerability is further compounded by cognitive immaturity, limited understanding of sexual boundaries, and social pressures, all of which increase susceptibility to grooming behaviors (6).

The literature also highlights the role of sociocultural factors in shaping grooming dynamics. Family environment, peer influence, and cultural norms may significantly affect vulnerability and disclosure processes. Children from socially isolated or emotionally neglectful environments may be at increased risk, while societal attitudes toward authority, sexuality, and victim disclosure can either hinder or facilitate protective interventions (7).

Technological developments have further expanded the traditional conceptualization of grooming. Digital platforms provide offenders with new opportunities to establish trust, manipulate emotions, and maintain ongoing interactions with victims. For example, instant messaging applications and social media platforms allow perpetrators to communicate continuously and privately, thereby facilitating prolonged and less detectable grooming processes (2).

Current research emphasizes that grooming is shaped by the interaction of individual, social, psychological, and technological factors. Victims’ personal characteristics, family dynamics, online behaviors, and broader social contexts intersect with perpetrators’ behavioral strategies, making grooming a multifaceted phenomenon that requires equally multidimensional prevention and intervention approaches. This integrative perspective highlights the importance of combining psychological, sociocultural, and technological frameworks in both future research and child protection practices.

## 4 Cyber grooming

Cyber grooming extends traditional grooming behaviors into online environments by leveraging digital affordances that increase accessibility, frequency, anonymity, and secrecy. Unlike offline grooming, which often requires physical proximity, cyber grooming enables offenders to communicate with victims across geographic boundaries, maintain continuous contact, and manipulate digital content to sustain emotional influence and control (8).

Cyber grooming generally follows stages similar to those observed in traditional grooming, including initial contact, rapport building, trust consolidation, sexualization, and eventual exploitation. However, digital communication may accelerate these processes through immediacy, anonymity, and persistent accessibility. Perpetrators may use fake identities, role-play as peers, and exploit children’s emotional and social needs to establish trust. Sexualized content is often introduced gradually to desensitize victims while reinforcing secrecy and emotional dependence.

The persistence and reproducibility of digital content may further intensify the psychological consequences experienced by victims. Images, videos, and chat records can be stored, redistributed, or used for coercion and extortion, contributing to prolonged trauma, anxiety, and fear of disclosure. The literature identifies several risk factors associated with cyber grooming vulnerability, including excessive online engagement, low digital literacy, social isolation, and inadequate parental supervision. Gender-related differences have also been reported, with girls more frequently targeted on social media platforms, whereas boys may be more vulnerable in gaming and forum-based environments (2, 9).

Technological infrastructure also plays a critical role in shaping grooming opportunities and risks. Platforms lacking effective age verification systems, moderation practices, or reporting mechanisms may inadvertently facilitate grooming behaviors. In contrast, proactive safety measures, platform-based monitoring systems, and accessible reporting tools may contribute to prevention and early detection efforts. These findings demonstrate the importance of integrating both technological and human-centered interventions in child protection strategies.

Current research emphasizes that cyber grooming is influenced by the interaction of developmental vulnerabilities, social context, family environment, online behaviors, and platform characteristics. Compared with traditional grooming, cyber grooming differs not only in communication medium but also in its scale, speed, accessibility, and persistence. Consequently, effective prevention and intervention strategies require multidimensional approaches that integrate psychological, social, technological, and educational perspectives. The key differences between traditional grooming and cyber grooming are summarized in **Table 1**.

**Table 1:** Key differences between traditional grooming and cyber grooming.

Dimension	Traditional grooming	Cyber grooming
Interaction	Face-to-face	Online (social media, messaging, gaming)
Reach	Geographically limited	Global, no spatial constraints
Speed	Gradual	Accelerated
Anonymity	Low	High
Victim Scope	Typically single	Multiple victims simultaneously
Control	Emotional manipulation	Emotional + digital coercion
Persistence	Episodic	Continuous (24/7)
Detection	Higher visibility	Lower visibility
Impact	Significant	Prolonged and intensified
Evidence	Physical/witness-based	Digital traces

## 5 Victim profile

The literature indicates that victims of grooming and cyber grooming are predominantly adolescents between the ages of 12 and 17, a developmental period characterized by identity formation, increased socialization, emotional sensitivity, and heightened online engagement (9). These developmental characteristics may increase vulnerability to manipulative behaviors that exploit emotional dependence, peer acceptance needs, and curiosity regarding sexual content and relationships.

Social and familial factors also play a significant role in shaping victimization risk. Children experiencing emotional neglect, inconsistent parental supervision, family conflict, or social isolation may be more susceptible to grooming behaviors. In addition, peer dynamics such as social comparison, peer pressure, and the pursuit of online validation can further increase vulnerability, particularly in digital environments where popularity and social approval are emphasized. Cultural attitudes toward authority, sexuality, and victim disclosure may likewise influence both vulnerability and reporting behaviors.

Gender-related patterns have also been identified in the literature. Girls are more frequently targeted on social media platforms, whereas boys may be more vulnerable within gaming environments and online forums. These differences appear to be associated with platform engagement patterns and gendered socialization processes. Despite contextual differences in victimization, both male and female victims may experience significant psychological consequences, including anxiety, depression, post-traumatic stress symptoms, low self-esteem, and social withdrawal (3).

The psychological impact of grooming may be intensified by delayed disclosure, secrecy, fear of stigma, and concerns regarding retaliation or social judgment. Early intervention approaches—including school-based education, psychological support services, parental involvement, and digital literacy programs—may help reduce vulnerability by improving awareness, strengthening resilience, and encouraging help-seeking behaviors. Furthermore, digital literacy education can enhance children’s ability to recognize manipulative interactions, establish personal boundaries, and report suspicious online behaviors.

Recent global evidence further demonstrates the magnitude of child sexual victimization worldwide. A systematic review and meta-analysis including 165 national-level studies from 80 countries found a lifetime prevalence of 11.4% for sexual harassment, 8.7% for contact sexual violence, and 6.1% for completed forced sexual intercourse. Girls reported higher rates of forced sexual intercourse than boys (6.8% vs. 3.3%), highlighting important gender-related differences in vulnerability and victimization experiences (10).

Overall, the reviewed literature demonstrates that victim vulnerability emerges through the interaction of developmental, psychological, social, familial, and technological factors. Rather than being determined by a single risk factor, susceptibility to grooming reflects the cumulative influence of multiple interconnected vulnerabilities. Consequently, effective prevention and intervention strategies require multidimensional approaches that integrate education, family engagement, psychological support, and platform safety measures to provide comprehensive protection for children and adolescents.

## 6 Perpetrator profile

The literature suggests that perpetrators involved in grooming and cyber grooming may exhibit psychological and behavioral characteristics such as empathy deficits, compulsive sexual interest in minors, cognitive distortions related to sexual entitlement, and a desire for dominance and control (6). These characteristics interact with social, situational, and technological factors, enabling offenders to manipulate victims and reduce the likelihood of detection.

Cyber grooming further increases perpetrators’ accessibility and operational reach. Online anonymity and reduced social accountability may enable offenders to contact multiple victims simultaneously while maintaining persistent communication over extended periods. Digital communication tools also facilitate emotional manipulation, coercion, and secrecy. Perpetrators may use flattery, emotional reinforcement, role-playing, or psychological pressure to establish trust and sustain control over victims.

Empirical evidence also suggests that online offenders may employ different approaches when initiating and maintaining contact with victims. In a qualitative analysis of naturally occurring online grooming interactions, Kloess et al. identified both indirect approaches characterized by rapport-building and gradual sexualization, and direct approaches involving immediate sexual communication. Interestingly, only a subset of offenders engaged in traditional grooming strategies, whereas others pursued more direct forms of sexual exploitation, highlighting the heterogeneity of offender behaviors in online environments (11).

The literature additionally highlights the influence of sociocultural and platform-related factors on grooming behaviors. Cultural norms that minimize awareness of child sexual abuse, tolerate inappropriate adult–child interactions, or stigmatize victim disclosure may contribute to environments that indirectly facilitate grooming. Similarly, poorly moderated digital environments or online communities that normalize sexualized interactions may increase opportunities for perpetration.

Understanding perpetrator characteristics is important for prevention efforts, risk assessment, and early intervention strategies. Identifying recurring behavioral, psychological, and technological patterns may assist professionals, educators, platform moderators, and law enforcement agencies in recognizing high-risk situations and improving child protection measures.

Current research emphasizes that effective prevention strategies require multidimensional approaches integrating psychological assessment, social context evaluation, and technological safety measures. This integrative perspective highlights the importance of examining perpetrator behaviors within broader situational and technological contexts to support more effective prevention and intervention frameworks.

## 7 Prevention, awareness, and legal frameworks

The literature emphasizes that effective prevention strategies for grooming and cyber grooming require comprehensive and multidisciplinary approaches integrating education, parental involvement, technological safeguards, and legal protections. Digital literacy programs may help children recognize manipulative behaviors, establish safer online practices, and report suspicious interactions (12). These interventions appear to be more effective when incorporated into school-based education and supported through family engagement.

Empirical evidence also supports the effectiveness of targeted educational interventions for preventing online grooming. In a randomized study involving 856 adolescents, a brief educational program on online grooming significantly reduced sexualized interactions with adults following online sexual solicitation and increased participants’ knowledge about grooming-related risks over a six-month follow-up period. These findings suggest that low-cost, school-based educational interventions may contribute to reducing vulnerability to online sexual exploitation and improving digital safety awareness among adolescents (13).

Parents and caregivers play a central role in prevention by supervising online activities, maintaining open communication, and promoting healthy interpersonal boundaries. Monitoring tools, content filtering systems, and accessible reporting mechanisms may further support protective efforts by reducing exposure to potentially harmful interactions. In addition, digital platforms that implement safety-oriented design principles, effective moderation practices, and responsive reporting systems may contribute to early identification and prevention of grooming behaviors.

Legal frameworks provide another important layer of protection in addressing online child sexual exploitation and abuse. Although Directive 2011/93/EU establishes obligations for the prevention and criminalization of child sexual abuse and exploitation, differences in enforcement practices and jurisdictional limitations continue to pose challenges. Therefore, international cooperation, harmonized legal procedures, and coordinated policy approaches remain essential components of effective child protection strategies in digital environments (14).

Beyond the European legal framework, international child protection instruments also play a critical role in addressing grooming and cyber grooming. The United Nations Convention on the Rights of the Child (CRC) establishes the obligation of States Parties to protect children from all forms of sexual exploitation and abuse and to implement appropriate preventive and protective measures (15). More recently, the United Nations Convention against Cybercrime has emphasized the importance of international cooperation, digital evidence sharing, and coordinated responses to technology-facilitated crimes affecting children in online environments (16).

The reviewed literature also highlights the importance of awareness campaigns targeting children, parents, educators, and communities. Educational initiatives that improve recognition of grooming behaviors, encourage disclosure, and clarify reporting pathways may contribute to reducing victimization risk. Collaboration among governmental institutions, non-governmental organizations, educational systems, and technology companies may further improve the reach and effectiveness of prevention efforts.

Overall, current evidence suggests that sustainable prevention requires coordinated interventions across educational, familial, technological, psychological, and legal domains. Effective child protection depends not only on individual awareness but also on broader systemic approaches that integrate social support, policy development, technological responsibility, and psychological resilience-building strategies. Strengthening these interconnected protective mechanisms is essential for supporting children’s safety and well-being in increasingly digitalized environments.

## 8 Discussion

The reviewed literature demonstrates that grooming and cyber grooming are complex and multidimensional phenomena shaped by the interaction of developmental vulnerabilities, social dynamics, and technological mediation. While traditional grooming primarily relies on face-to-face interactions to establish trust and emotional dependence, cyber grooming utilizes digital affordances such as anonymity, immediacy, and persistent connectivity to facilitate access to minors and prolong engagement (1, 5). Digital environments not only expand perpetrators’ accessibility beyond geographic limitations but may also enable simultaneous interactions with multiple victims while reducing opportunities for direct supervision and detection.

The psychological mechanisms underlying grooming behaviors appear to remain relatively consistent across offline and online contexts; however, digital environments may intensify their impact. The literature suggests that offenders frequently exploit adolescents’ developmental needs for social connection, emotional validation, and identity formation while reinforcing secrecy and emotional dependency through ongoing digital communication. Studies examining cyber grooming behaviors describe recurrent strategies including rapport building, emotional manipulation, exploitation of psychological vulnerabilities, and maintenance of secrecy, all of which may contribute to prolonged victimization and delayed disclosure (17).

These findings suggest that cyber grooming may be understood within a developmental framework in which normative adolescent processes, including identity exploration, peer affiliation, and emotional validation seeking, can be exploited by offenders. This perspective highlights the importance of considering cyber grooming not only as a form of online victimization but also as a process that intersects with key developmental and psychological challenges during adolescence.

Victim vulnerability appears to be multifactorial and influenced by the interaction of psychological, social, familial, and technological factors. Adolescents with high levels of online engagement, limited digital literacy, reduced parental supervision, or insufficient offline social support may be at increased risk of cyber grooming. Existing epidemiological evidence indicates that online sexual solicitation and grooming-related interactions remain prevalent among adolescents. In addition, behaviors such as sexting, running away from home, and exposure to coercive threats have been associated with increased vulnerability to more severe forms of online sexual exploitation, with female adolescents frequently overrepresented in reported cases (18).

The reviewed evidence further indicates that perpetrator behaviors are influenced not only by psychological characteristics but also by situational and technological contexts. Empirical studies involving individuals engaged in online grooming behaviors suggest diverse motivational pathways, including compulsive tendencies, social or emotional needs, fantasy-related motivations, and role-playing behaviors (11). Furthermore, online environments may reduce perceived social consequences and lower behavioral inhibition, thereby complicating prevention and intervention efforts.

Technological affordances present both risks and opportunities in the context of cyber grooming. Platforms lacking adequate moderation systems, age verification procedures, or accessible reporting tools may inadvertently facilitate grooming behaviors. At the same time, emerging approaches involving automated detection systems, message-level analysis, and proactive moderation strategies may support earlier identification of potentially harmful interactions. Nevertheless, ethical considerations, privacy concerns, and implementation limitations remain important challenges within technology-assisted prevention efforts (19).

The reviewed literature emphasizes that effective prevention strategies must be multidimensional and multidisciplinary. Digital literacy education that includes not only technical competence but also critical awareness of online social manipulation may help reduce vulnerability. Family engagement, open communication regarding online experiences, and supportive supervision remain central protective factors. In addition, policy responses promoting safety-oriented platform design, accessible reporting systems, and coordinated legal protections may strengthen child safety in digital environments. These efforts should be supported by international legal instruments, including the Convention on the Rights of the Child and emerging cybercrime frameworks that facilitate cross-border cooperation in the prevention and investigation of online child sexual exploitation.

Despite growing research interest, important gaps remain in the literature. Longitudinal studies evaluating the long-term psychological and social consequences of cyber grooming remain limited, and prevalence estimates continue to vary considerably across methodologies and geographic regions. For example, a recent global meta-analysis reported a pooled prevalence of 8.1% for overall online child sexual exploitation and abuse, while prevalence estimates for specific forms of online solicitation and image-based victimization exceeded 12%, highlighting substantial variation across definitions, methodologies, and study populations (4). Further research is also needed to evaluate the effectiveness of prevention programs integrating psychological, educational, technological, and social interventions. Moreover, rapidly evolving digital ecosystems including encrypted communication platforms, immersive virtual environments, and AI-mediated interactions require ongoing investigation to identify emerging grooming modalities and develop responsive child protection strategies.

The findings of this review should be interpreted within the context of the broader body of evidence available in the current literature. Although only studies meeting the predefined eligibility criteria were included in this scoping review, the findings of large-scale meta-analyses encompassing 123 and 165 studies, respectively, demonstrate broadly consistent trends. Comparison of our findings with these comprehensive evidence syntheses indicates that the conclusions of the present review are largely consistent with the existing international literature, while acknowledging certain contextual differences that may arise from variations in study populations, intervention characteristics, and healthcare settings. Placing our findings within the context of these large-scale syntheses strengthens the interpretation of the results and more clearly positions the present review within the existing evidence base.

## 9 Implications for psychology

The findings of this review have several implications for psychology research and practice. Greater attention should be directed toward understanding the psychological mechanisms that contribute to vulnerability in online environments, including emotional needs, social isolation, risk-taking behaviors, and developmental factors. In addition, psychologists, school counselors, educators, and mental health professionals may play an important role in identifying at-risk individuals, promoting digital resilience, and supporting prevention efforts through evidence-based interventions and psychoeducational programs.

Overall, current evidence indicates that cyber grooming should be understood not solely as a technological issue but as a broader psychological, social, and public health concern requiring coordinated multidisciplinary responses. Effective prevention depends on integrating individual-level education, family and community engagement, technological responsibility, and systemic child protection measures. Strengthening digital competence, improving social support systems, and implementing evidence-based protective strategies may collectively contribute to reducing grooming-related risks and enhancing children’s safety and well-being in digital environments.

## 10 Conclusion

Cyber grooming represents a multidimensional form of exploitation involving psychological manipulation, developmental vulnerability, social influences, and technological facilitation. The reviewed literature suggests that cyber grooming should be understood not only as a digital safety concern but also as a developmental and psychological phenomenon that exploits normative adolescent needs for social connection, emotional validation, and identity formation. Effective child protection therefore requires comprehensive approaches integrating digital literacy education, family involvement, psychological support, platform safety measures, and legal protections.

Children and adolescents should be supported in developing critical digital awareness and safe online behaviors, while caregivers and educators play essential roles in supervision, communication, and early intervention. In parallel, digital platforms and policymakers should continue to strengthen safety-oriented practices, accessible reporting systems, and cross-jurisdictional legal cooperation to address the evolving nature of online exploitation.

The findings of this scoping review further highlight the need for longitudinal research examining long-term psychological outcomes, the effectiveness of prevention strategies, and the influence of emerging technologies on grooming behaviors and victimization patterns. As digital environments continue to evolve, sustainable child protection will require coordinated, evidence-based, and multidisciplinary responses that integrate psychological, social, educational, technological, and legal perspectives.

Ultimately, protecting children in digital environments is a shared societal responsibility requiring ongoing collaboration among families, educators, healthcare professionals, policymakers, technology developers, and law enforcement agencies to promote children’s safety, resilience, and well-being.
